# Nervous Diseases

**Published:** 1891-05

**Authors:** James W. Putnam


### Progress in Medical Science.

### NERVOUS DISEASES.

Conducted by JAMES W. PUTNAM, M. D.

PITTS (*University Medical Magazine*) has added to the list of cases of Pressure Paralysis the following case of palsy of the deltoid muscle, caused by pressure upon the circumflex nerve. This is the only case of the kind in literature. The history is briefly as follows :

T. T., aged 40, single, and a blacksmith by occupation, came to the Nervous Dispensary complaining that four weeks before, after drinking heavily all day, he went to bed and on awakening the following morning, found that "he could not lift his left arm above his head."

On examination it was found that the patient could elevate the arm only to a very slight degree in any direction. All other movements could be performed without difficulty. The deltoid was flabby and reactions of degeneration were present. There was no pain and no tenderness on pressure over any of the nerve trunks ; sensation was not affected.

Under the use of strychnia and galvanism he made a good recovery in a few weeks.

As to the cause of this palsy, it is probable that he laid upon his back so close to the side of the bed that the edge of the sideboard exerted continuous pressure upon the circumflex nerve where it winds posteriorly around the neck of the humerus.

### IS CHOLERA A NEUROSIS ?

CHOLERA is ever an interesting disease, and the theory laid down by Alex. Hackin, F. R. C. S., that it is a neurosis, whatever its origin may be, has led him to adopt an entirely new treatment, which is ably sustained in the *Medical Age*. He believes all the phenomena to be due to the troubles produced in the functions manifested in the sympathetic system. He says the vomiting and the numerous stools evidently are connected with the attack upon the nervous apparatus of the stomach and the intestines ; the choleric crisis, the cramps, the vertigo, the anxiety, the spasms, the tremblings, have a nervous origin. It is to the vaso-motors, then, that we must refer the depression of the functions of respiration and circulation, the gravest symptoms in cholera.

With this idea he treated cases of English cholera by using the antagonism of the pneumogastric nerves as the means of checking the action of the sympathetic nerves. In every case, he observed that by stimulating the pneumogastric in the neck, or by developing the inhalitory power, the vomiting, the stools, and the cramps were arrested. The simplicity of the treatment at once attracts us. In all cases of cholera, whatever the stage, he applies with a brush the epispastic liquor of the B. P. on the branches of the pneumogastric in the neck, or the mastoids, or under the eye, covering about three inches of surface. The effect is generally instantaneous.

As an illustration we will quote a case reported by Dr. Cannatacci, at the hospital at Zeitum. He says the treatment of Dr. Hackin is the most useful in asiatic cholera.

At half-past eight I was called to see policeman Banack. When I arrived I found the following phenomena :

Vomiting, feeble pulse, violent cramps, palpitation of the heart, great debility, coldness of the extremities, numerous rice like stools. He told me that on arriving at the barracks he had been seized with profuse vomiting, then, after the lapse of a half hour, with numerous stools and cramps.

The attacks succeeded each other every quarter hour. I administered no medicine, but applied the epispastic liquor under both eyes, assuring him that none of his symptoms would return. I saw him at half-past ten ; he was convalescing, and had had no more passages or attacks of vomiting.

---

### THE TREATMENT OF EPILEPSY.

The treatment of epilepsy being so unsatisfactory, it continues to invite new therapeutic measures. Among recent suggestions we notice in the *Journal of the American Medical Association*, that bromide of gold has been employed in Russia, Germany and Belgium, as a nervine and anti-epileptic. Dr. Goubart, of Brussels, has reported that, in a certain proportion of cases, it has been better borne that any of the bromides. According to him, the initial dose for adults should be one-eighth of a grain, to be increased gradually to one-fifth ; for children, from one-twentieth to one-tenth of a grain. The drug is a yellowish gray friable mass, *insoluble in water*. Dr. Pott's reports results of treatment suggested by Dr. H. C. Wood, of Philadelphia, some years ago : "In all cases when antipyrin and bromide of ammonium were used, there was marked relief of the symptoms, and in some cases when other remedies had failed to give relief. There were no indications of bromism and none of the unfavorable symptoms of antipyrin observed. Six grains of antipyrin were usually given *with twenty of the bromide.*"

---

### NERVE-GRAFTING.

IN THE *Review of Insanity and Nervous Diseases*, we find that Atkinson reports a number of successful cases of nerve-grafting to the British Medical Association, September 13th. It is important to bear in mind that the substitution of a new piece of nerve for a piece that has been lost (nerve-grafting) is a totally different thing from the uniting of a nerve (nerve-suturing). In one case the median nerve had been divided in an operation. Two and one-half inches of the tibial nerve from an arm that happened to be amputated at the same time, was transplanted to the forearm of his patient. Healing occurred by first intention, and sensation began to return in thirty-six hours, and in five weeks was complete. The muscles partly recovered their power. A number of other cases were reported with similar results. Strict asepis is important, though union may occur following suppuration. Return of sensation occurs before motion. Better results are obtained when the grafting is performed *immediately after the injury.*

---

### RECENT LEGISLATION FOR THE INSANE IN THE STATE OF NEW YORK.

DR. CARLOS F. MACDONALD, the President of the State Commission in Lunacy, recently read a paper with the above title in New York City, in which he states that for all this class of dependants, a more enlightened and humane system of treatment and care will be secured, and greater protection will be afforded against possible abuse and wrong, in their commitment, custody and control. The legislation referred to embraces the following :

1st. Providing for extension of State hospital accommodations.

2d. Creation of a State Commission in Lunacy.

3d. Providing for a removal of the insane from county poor-houses to State hospitals.

4th. Changing the corporate title of the respective State institutions for the insane, to that of State hospitals, thus recognizing and establishing the hospital idea.

5th. Providing for the employment of a female physician at each State hospital, as an adjunct to the regular medical staff.

The creation of the State Commission in Lunacy was to provide for an independent governmental supervision of the insane, and of all institutions devoted to their custody. He proceeds then, in detail, to explain the twenty-one sections of the act, from which he concludes that the supervisory and correctional powers of the Commission, as regards the insane and the management of institutions, are practically unlimited. He reviews the work of the Commission for the last eighteen months, and demonstrates, very clearly, that the Commission has already done much for the improvement of this unfortunate class.

---

A NOVEL experiment is about to be tried in the treatment of insomnia, in the department for men in the State Hospital for Insane of the south-east district of Pennsylvania. Dr. Chase says he is about to begin a series of experiments in the treatment of insanity in rythmic sound, based on the principle involved in the treatment of nervous diseases, with rythmic flashes of light. The basis on which he proposes to experiment, includes those suffering from insomnia. He will use a mechanism similar to an electric drum, when rythmic vibrations can be completely controlled both in velocity and pitch. He believes that a pleasant sound, by the means suggested, pitched just above the irregular sonorific disturbances of turning in bed, coughing, snoring, sighing, etc., of a dormitory or ward, might be attended with beneficial results; or happily administered in individual cases.

The novelty of the method strikes us, but we cannot say that it commends itself at first glance.

